# Artificial intelligence for tuberculosis management in Africa: opportunities, challenges, and implementation

**DOI:** 10.3389/fpubh.2026.1816634

**Published:** 2026-05-20

**Authors:** Imane Chaoui, Mohammed Amine Koulali, Onesime Mbulayi, Saint-Jean Djungu, Loukia Aketi, Abdou Azaque Zouré, Jamaleddine Bourkadi, Yahya Tayalati, Mohammed El Mzibri, AI4TB Consortium

**Affiliations:** 1 Department of Life Sciences, Centre National de l’Energie, des Sciences et des Techniques Nucléaires (CNESTEN), Rabat, Morocco; 2 Laboratoire de Modélisation et Calcul Scientifique (LMCS), ENSAO, Mohammed First University, Oujda, Morocco; 3 College of Computing, Mohammed VI Polytechnic University, Ben Guerir, Morocco; 4 Department of Mathematics, Statistics and Informatics, University of Kinshasa, Kinshasa, Democratic Republic of Congo; 5 Cardio-Pulmonary and Infectious Diseases Unit, Department of Pediatrics, University of Kinshasa, Kinshasa, Democratic Republic of Congo; 6 Institut de Recherche en Science de la Santé, Centre National de Recherche Scientifique et Technologique (IRSS/CNRST), Ouagadougou, Burkina Faso; 7 Pneumology Department, Moulay Youssef Hospital - Ibn Sina University Hospital, Rabat, Morocco; 8 Department of Physics, Faculty of Sciences, University Mohammed V, Rabat, Morocco; 9 School of Applied and Engineering Physics, Mohammed VI Polytechnic University, Ben Guerir, Morocco

**Keywords:** accurate diagnosis, Africa, artificial intelligence, prediction, tuberculosis

## Abstract

**Objectives:**

Worldwide, tuberculosis (TB) continues to be an important cause of human morbidity and mortality, particularly in developing countries where drug surveillance and rapid detection of resistance to anti-TB drugs is uncommon and the lack of proper healthcare systems often leads to incomplete treatment and spread of MTB strains. The present paper discusses the potential role of artificial intelligence (AI) in improving TB diagnosis, management, and control in African countries, where conventional diagnostic approaches remain predominant and often insufficient.

**Methods:**

A narrative review of current challenges in TB management in African settings was conducted, focusing on limitations of existing diagnostic tools and the emerging contributions of AI-based technologies in healthcare. The review demonstrates how machine learning algorithms and computer-aided systems could be integrated into TB programs to enhance clinical decision-making and surveillance.

**Results:**

Conventional TB diagnostic methods such as the tuberculin test, radiography, and microscopic examination show limited accuracy and efficiency, contributing to ongoing transmission and poor treatment outcomes in many African countries. AI innovations have demonstrated improved performance in disease detection and prediction across various health domains, offering time-saving, resource-efficient, and scalable solutions. Applied to TB, AI could support clinicians in diagnosis, forecast treatment outcomes, and strengthen public health strategies aimed at controlling MTB spread.

**Conclusion:**

AI holds significant promise for enhancing TB control efforts in African countries by improving diagnostic precision, clinical decision-making, and surveillance capacities. Integrating AI into national TB programs could promote more effective and efficient disease management, ultimately contributing to reduced morbidity and mortality.

## Background

Tuberculosis (TB), caused by *Mycobacterium tuberculosis* (MTB) continues to be a significant public health challenge with high morbidity and mortality rates worldwide. It is estimated that one-quarter of the global population is latently infected with *M. tuberculosis* and leads to 2 million deaths annually of which 90% occur in developing countries (1). Despite the world Health Organization (WHO) End TB Strategy, which aims to reduce TB incidence by 90% and deaths by 95% by 2035, progress remains insufficient, with only a 23% decline in mortality since 2015 (2).

Notably, national TB programs in Africa face numerous obstacles in their fight against TB. The major issues are limited laboratory capacity, delayed diagnosis, insufficient drug surveillance, which contributes to disease propagation, and even when cases are identified, ensuring patient adherence to treatment remains a significant obstacle, often leading to treatment failure. Over the past three decades, there has been a dramatic rise in the resistance and spread of multidrug-resistant (MDR) strains, which are resistant to the two frontline drugs, isoniazid (INH) and rifampicin (RIF). Subsequently, a more concerning scenario emerged for TB control programs with the discovery of extreme drug resistant TB (XDR-TB) (3), raising fears of a future epidemic of virtually untreatable TB (4). The development of these resistant TB forms presents new hurdles for TB control programs in Africa particularly in the context of co-epidemics with HIV and/or malaria. These poverty-related diseases are considered overlapping epidemics, which complicate individual case management and overall effectiveness of control programs for all three diseases (5).

Despite the diligent efforts made by TB national programs, tuberculosis remains a significant health priority in many African countries. TB infection is closely linked to social and economic determinants such as malnutrition, diabetes, HIV, alcohol use and tobacco consumption. Thus, an effective fight against TB requires coordinated, accountability, and multi-sectoral actions, that must be implemented in a timely manner to ensure accuracy and efficiency. Furthermore, the COVID-19 Crisis has hindered TB case detection efforts by diverting financial resources and support toward the fight against COVID-19 further exacerbating the challenges faced in combating tuberculosis. Conventional diagnostic tools remain widely used despite their limitations, contributing to ongoing transmission and suboptimal care. In this context, artificial intelligence (AI) offers promising opportunities to enhance TB control through improved diagnostics, clinical decision-making, and surveillance, particularly in resource-limited settings.

This manuscript is based on a narrative review of the literature, aiming to review the current challenges in TB diagnosis and management in African countries, and to explore the potential role of AI-based technologies in strengthening TB control and healthcare delivery.

A comprehensive search was conducted in PubMed, Scopus, and Google Scholar for publications from January 2015 to December 2025, using the following keywords: “tuberculosis,” “artificial intelligence,” “digital chest X-ray”, “drug-resistant TB,” and “Africa.” Boolean operators were applied to refine the search, using combinations such as:“tuberculosis” AND “artificial intelligence,” “tuberculosis” AND “digital chest X-ray” AND “Africa”, “drug-resistant tuberculosis” AND “artificial intelligence,”

Publications were eligible for inclusion if (i) they were peer-reviewed research articles or systematic reviews, or reports from the WHO; (ii) addressed tuberculosis diagnosis, treatment monitoring, drug resistance, or surveillance; (iii) examined applications of AI in TB management; (iv) focused on African countries, and (v) were published in English or French. Peer-reviewed articles and relevant WHO reports were screened and synthesized.

## Challenges in TB diagnosis and drug resistance profiling

Currently, despite the availability of new molecular diagnostic approaches, many low- and middle-income countries still rely on conventional diagnostic approaches with well documented limitations. These approaches include:

- Tuberculin test: This test is widely used to identify individuals infected with TB bacteria. This test is not sufficient and additional tests are needed to confirm TB and to distinguish between latent TB infection or TB disease.
- Radiographic imaging (Chest X-ray): While chest X-ray is more sensitive than clinical symptom enumeration, its use in TB screening is limited due to high inter- and intra-reader’s variability, modest reproducibility among human readers and the scarcity of radiologists in high-burden African countries.
- Microscopic examination: Many African countries still rely on smear microscopy, which is less sensitive than the rapid diagnostic tests recommended by the WHO, making a gap between the estimated global burden of TB cases and the number of cases actually detected and notified.

Consequently, the limited ability to identify patients with TB contributes to the ongoing rise in disease transmission in developing countries and its re-emergence in developed ones. Over the past few decades, the management of TB has nevertheless undergone significant conceptual and methodological progress, leading to key improvements in early diagnosis, drug resistance detection, treatment regimen, and monitoring strategies for better TB management (6). In response to these challenges, many African countries, either independently or with WHO support, have made efforts to ensure early diagnosis and effective treatment (7). Accordingly, recent advances in film-screen radiography systems have also enhanced the quality of standard chest radiographs by incorporating automated, robust image-processing techniques that deliver excellent image quality and consistent results (8).

## Molecular tools for TB diagnosis and drug resistance profiling

In recent years, globalization has facilitated the adoption of advanced technologies within African health systems, enabling the implementation of WHO-recommended rapid molecular tests for TB detection and drug-resistance testing, including rapid molecular tests such as Xpert MTB/RIF and Xpert MTB/RIF Ultra, WHO-endorsed line probe assays (LPAs), and Loop-Mediated Isothermal Amplification (TB-LAMP), and, in specialized reference laboratories, whole-genome sequencing (9). Their high sensitivity and specificity, and the short time required to perform the procedure have fuelled their widespread adoption in routine diagnostic laboratories and the enthusiasm of health providers to incorporate these methods in the overall management guidelines (10). The interest in using molecular techniques in clinical microbiology has grown significantly following the COVID-19 pandemic, which accelerated the upgrading of medical laboratories and the broader implementation of molecular analyses.

Another critical dimension of TB control concerns the transmission dynamics of MTB strains. In this context, molecular epidemiology has greatly enhanced our understanding of TB transmission and has complemented conventional epidemiologic methods in the investigation of outbreaks, laboratory cross-contamination, distinction between recent transmission and reactivation of latent TB and providing valuable insights to delineate risk factors for the recent acquisition of MTB infection and progression to TB disease (11). The adoption of advanced molecular techniques and technologies is becoming crucial in the era of globalization, where heightened human mobility and increased population dynamic contribute to the rapid spread of bacterial pathogens and infectious diseases across regions and international borders (12).

Therefore, to address persistent diagnostic challenges, particularly in resource-limited settings with high TB incidence and where there is a shortage of radiologists, the integration of artificial intelligence (AI) solutions holds significant promise for TB detection and clinical decision support. AI-based systems can assist in interpreting X-rays for TB diagnosis, thereby enhancing early detection and facilitating timely and accurate treatment in developing countries. Notably, chest X-rays can be captured using a simple cell phone camera and transmitted for remote analysis by AI algorithms (13). Studies have demonstrated that smartphone-captured photographs of chest radiographs can be processed by artificial intelligence models with comparable performance to traditional digital images, enabling remote interpretation and diagnostic support in resource-limited settings. Consequently, AI software may prove to be as effective as a qualified radiologist to bridge this gap for earlier TB detection and improved disease control (14).

## The contribution of AI for TB management in Africa

Universal access to drug sensitivity testing remains a major challenge, yet early identification of drug-resistant cases, including MDR-TB and XDR-TB, significantly enhances the likelihood of successful treatment and helps interrupt transmission. In this context, the development of AI-powered predictive models for multidrug-resistant pulmonary tuberculosis is crucial for timely treatment, preventing the spread of drug-resistant TB, and limiting the emergence of more severe forms of resistance (15).

For long time, the African continent was often perceived as significantly lagging behind in technological advancement. While adoption of modern techniques may have been slower, there is now an increasing recognition among African nations of the potential benefits of leveraging recent technological advancements to improve population health. Similar to other sectors, there is an urgent need to transform healthcare in African countries by integrating new digital tools, such as shared medical records, harnessing Big Health Data and utilizing AI to enhance diagnosis, support health decision-making and elaborate predictive models. In this regard, AI models have been widely proposed as promising solutions to bridge the gap between patient needs and available resources and infrastructures. These innovative digital tools are increasingly recognized for their ability to enhance diagnosis in resource-limited settings (16), to enhance drug resistance screening for better management of the disease (17) and to increase the control of strains spread to prevent further dissemination of virulent strains or those with particular drug resistance patterns (18). Moreover, AI holds the potential to optimize healthcare professionals’ workloads, leading to substantial resource savings that can be redirected toward research, innovation and/or infrastructure improvement initiatives.

## Tentatives in the use of AI for TB in African countries

Over the past decade, numerous initiatives have been launched in African countries to adopt AI in various infectious disease control, in line with the goals set by the WHO. These are examples of AI and machine learning initiatives in TB management across different regions:

1. Deep learning for chest radiography in Cameroon, which offers a scalable adjunct to conventional TB screening, especially in low-resource, high-burden settings (19).
2. AI models for TB incidence forecasting in Eastern Cape, South Africa, supporting enhanced surveillance, planning, and TB control (20).
3. ARIMA and hybrid ARIMA–ANN models in Kenya to predict TB cases among children using routine surveillance data (21).
4. AI-guided treatment optimization for pulmonary MDR-TB in Tanzania, identifying pharmacokinetic-pharmacodynamic indices linked to clinical outcomes highlighting the potential of AI to inform precision medicine approaches (22).
5. In Nigeria and Zambia, AI-based computer-aided detection (CAD) systems have been used to enhance TB screening thereby improving diagnostic efficiency (23, 24).
6. AI-driven approaches have also been applied in Ethiopia to optimize diagnostic workflows and enhance clinical decision-making using available data from laboratory records (25).

Collectively, these initiatives underscore AI’s versatility, from point-of-care screening to public health forecasting, and emphasize the importance of huge datasets, good technical infrastructure and local expertise. Implementing AI-driven approaches for precise drug resistance prediction, real-time monitoring of TB strain circulation, and tracking transmission dynamics of tuberculosis is crucial in high burden African countries and is key to achieving WHO End TB Strategy targets.

Collaboration between national and international interdisciplinary research networks will be crucial to the successful implementation of AI techniques, given the complex nature of the disease and diverse factors influencing its spread and treatment. In this context, AI techniques, particularly, machine learning algorithms can enhance TB detection and treatment guidance, distinguish between drug sensitive from drug resistant patterns, predict outcomes, and enable real-time monitoring of TB spread, facilitating prompt and effective responses to outbreaks. However, substantial knowledge gaps persist regarding the most effective ways to implement these approaches in resource-limited regions.

It is also imperative to evaluate the diagnostic capacity and accuracy of the AI models compared to pre-existing rapid molecular tests and gold standard methods, such as culture and drug susceptibility testing (DST). While DST remains the reference standard, it is time-consuming and costly. AI, leveraging data mining and modeling, offers a promising approach for classifying potential cases of drug resistance based on the combination of multiple factors namely clinical history, genomic markers, demographic data, and radiological features. It is expected that AI models may yield diagnostic capabilities comparable to, or even surpassing, those of rapid molecular tests. Additionally, the cost-effectiveness of the IA model should be assessed against standard diagnostic tools.

In Africa, despite extensive efforts to reduce inequality, alleviate poverty, improve health management and support economic development, many countries still face socio-economic challenges, outdated policies, and limited focus on technological innovation.

Globally, in the last decade, guidelines recognizing the value of integrating AI models to enhance customer experience, improve operational efficiency and generalize automate processes have been established in most developed countries, trends now being applicated in developing countries, including Africa. The growing adoption of AI is driven by its numerous benefits in data management, supporting decision-making, and predicting risks across various socioeconomic sectors, with a particular focus on human health. The use of AI in TB management in Africa offers numerous advantages, most importantly:

1. Early diagnosis to timely initiate treatment which will reduce infectiousness period and improve patient outcome.
2. Prompt case notification to public health authorities.
3. Differentiation of acid-fast bacillus (AFB) smear positive specimens from other Mycobacteria can avoid unnecessary contact investigations.

## Recommendations for successful implantation of AI

To ensure the successful implementation of AI in African countries and maximize its benefits for national TB programs, several key considerations must be addressed:

1. Development of an integrated and efficient approach that combines AI technologies with existing TB diagnostic protocols, suitable for both routine use and specific clinical or programmatic scenarios.
2. Strict adherence to ethical principles governing in AI for TB prevention, diagnosis, treatment, and monitoring.
3. Ensuring equitable and universal access to AI-based tools so that all populations benefit without exclusion or discrimination.
4. Careful assessment and mitigation of potential risks associated with AI implementation, for both patients and national TB programs.
5. Promotion of accountable AI use that primarily supports, and in certain contexts appropriately substitutes, human decision-making while maintaining transparency and oversight.
6. Establishment of a pan-African research program to promote collaboration among interdisciplinary researchers, fostering the development, testing, and deployment of innovative AI-based solutions for effective TB surveillance and management, ultimately

## Limitations and future prospects

Despite the growing potential of AI in TB management in Africa, major challenges still remain. These include scarce high-quality datasets, limited digital infrastructure and insufficient technical expertise. Future progress will depend on adapting global lessons to local contexts: COVID-19 and malaria AI initiatives demonstrate the value of real-time data sharing, interoperable systems, and international collaboration (26, 27). Widespread adoption of AI also requires robust legal and ethical safeguards, including data protection, regulatory clarity, and algorithmic transparency.

## Conclusion

To sum up, integrating AI into TB management in Africa offers significant potential to address the challenges faced by healthcare systems in the region. AI can enhance TB care at various stages, but overcoming challenges related to infrastructure, data and acceptance is crucial for realizing its full potential.
